# The Effects of NF-κB and c-Jun/AP-1 on the Expression of Prothrombotic and Proinflammatory Molecules Induced by Anti-β_2_GPI in Mouse

**DOI:** 10.1371/journal.pone.0147958

**Published:** 2016-02-01

**Authors:** Longfei Xia, Hongxiang Xie, Yinjing Yu, Hong Zhou, Ting Wang, Jinchuan Yan

**Affiliations:** 1 Jiangsu Key Laboratory of Medicine Science and Laboratory Medicine, School of Medicine, Jiangsu University, Zhenjiang, Jiangsu Province, China; 2 Department of Cardiology, Affiliated Hospital of Jiangsu University, Zhenjiang, Jiangsu Province, China; Universidade de São Paulo, BRAZIL

## Abstract

Our previous data demonstrated that nuclear factor-κB (NF-κB) and activator protein-1 (AP-1) are involved in the process of anti-β_2_GPI/β_2_GPI-induced tissue factor (TF) expression in monocytes. However, the role of NF-κB and AP-1 in pathogenic mechanisms of antiphospholipid syndrome (APS) *in vivo* has been rarely studied. This study aimed to investigate whether NF-κB and c-Jun/AP-1 are involved in anti-β_2_GPI-induced expression of prothrombotic and proinflammatory molecules in mouse. IgG-APS or anti-β_2_GPI antibodies were injected into BALB/c mice in the presence or absence of PDTC (a specific inhibitor of NF-κB) and Curcumin (a potent inhibitor of AP-1) treatment. Our data showed that both IgG-APS and anti-β_2_GPI could induce the activation of NF-κB and c-Jun/AP-1 in mouse peritoneal macrophages. The anti-β_2_GPI-induced TF activity in homogenates of carotid arteries and peritoneal macrophages from mice could significantly decrease after PDTC and/or Curcumin treatment, in which PDTC showed the strongest inhibitory effect, but combination of two inhibitors had no synergistic effect. Furthermore, anti-β_2_GPI-induced expression of TF, VCAM-1, ICAM-1 and E-selectin in the aorta and expression of TF, IL-1β, IL-6 and TNF-α in peritoneal macrophages of mice were also significantly attenuated by PDTC and/or Curcumin treatment. These results indicate that both NF-κB and c-Jun/AP-1 are involved in regulating anti-β_2_GPI-induced expression of prothrombotic and proinflammatory molecules *in vivo*. Inhibition of NF-κB and c-Jun/AP-1 pathways may be beneficial for the prevention and treatment of thrombosis and inflammation in patients with APS.

## Introduction

The antiphospholipid syndrome (APS) is an acquired autoimmune disease characterized by the occurrence of thrombosis and/or recurrent miscarriages. APS is also associated with the presence of antiphospholipid antibodies (aPL), including lupus anticoagulant (LA), anticardiolipin antibodies (aCL) and anti-β_2_-glycoprotein I antibodies (anti-β_2_GPI) [1–2]. There is a close relationship between aPL and vascular thrombotic events and an array of obstetric complications [3]. In APS, most aPLs are autoantibodies directly against phospholipid binding proteins such as β_2_GPI and prothrombin [4]. The majority of studies have demonstrated that β_2_GPI is the major antigenic target, and anti-β_2_GPI autoantibodies are predominantly responsible for the clinical manifestations of APS [5]. High titers of anti-β_2_GPI antibodies are frequently found in the plasma of the patients, suggesting their important roles in APS [6].

The pathogenic mechanisms of APS have not been fully elucidated, and a number of mechanisms have been proposed. Increasing evidence has indicated that aPL/anti-β_2_GPI may bind to cells via its specific binding molecules/receptors, such as annexin A2 (ANX2) and Toll-like receptor 4 (TLR4), causing the activation of endothelial cells (ECs), monocytes and platelets [7–9]. Some Studies have also suggested that aPL/anti-β_2_GPI can recognize β_2_GPI binding to monocytes and ECs, leading to a series of alterations in intracellular signaling pathways *in vitro*. These alterations can result in prothrombotic and proinflammatory phenotypes of monocytes and ECs, e.g., secretion of inflammatory cytokines (IL-6, IL-1β, TNF-α, and IL-8), induction of adhesion molecules (VCAM-1, ICAM-1 and E-selectin) and TF [10–11]. Moreover, aPL/anti-β_2_GPI may cause thrombosis, ECs activation and pregnancy loss in animal models of APS [12–14]. Furthermore, a multiple of studies illuminated that TLR4/NF-κB pathway is involved in mediating the pathogenic effects of aPL/anti-β_2_GPI on ECs and monocytes [15–16]. Our previous results implicated that both nuclear factor-κB (NF-κB) and activator protein-1 (AP-1) are involved in anti-β_2_GPI/β_2_GPI-induced TF expression in monocytes [17]. However, whether NF-κB and AP-1 are activated *in vivo* or whether inhibitors of NF-κB and AP-1 are effective in reversing prothrombotic and proinflammatory effects of aPL/anti-β_2_GPI *in vivo*, have not been well studied.

NF-κB is one of the key cytoplasmic transcription factors and expressed in almost all cell types to mediate the expression of more than 100 different genes. Numerous NF-κB target genes are relevant to immune responses and inflammation [18]. NF-κB is a complex of heterodimeric and homodimeric transcription factors, and NF-κB family is composed of RelA, c-Rel, RelB, NF-κB1 (p50 and its precursor p105), and NF-κB2 (p52 and its precursor p100) [18]. NF-κB is normally kept inactive in the cytoplasm by interaction with IκBs. Upon stimuli (such as inflammatory cytokines, infection with viruses or LPS, stress signals), IκBs are phosphorylated and degraded by ubiquitin-proteasome pathway, leading to the activation of NF-κB. Activated NF-κB can enter into nucleus to regulate the transcription of target genes [19]. Transcription factor AP-1 consists of heterologous dimeric complex that contains members of the JUN, FOS, ATF and JDP subunits. The different AP-1 factors may regulate different target genes through interaction with specific protein kinases and a variety of transcriptional coactivators, and thus execute distinct biological functions such as cell proliferation and survival [20]. Generally, the main ingredients of AP-1 are c-Jun and c-Fos in mammals. Similarly, the activity of NF-κB and AP-1 can be regulated by several upstream kinases, such as p38-mitogen-activated protein kinase (p38 MAPK), extracellular signal-regulated kinase (ERK) and c-Jun N-terminal kinases (JNKs) [21].

However, whether both NF-κB and c-Jun/AP-1 are involved in aPL/anti-β_2_GPI-induced expression of TF, adhesion molecules and inflammatory cytokines *in vivo* has not yet been clarified. Taking into account these circumstances, we investigated the effects of NF-κB and c-Jun/AP-1 on aPL/anti-β_2_GPI-induced expression of prothrombotic and proinflammation molecules by using a specific NF-κB antagonist pyrrolidinedithiocarbamate acid (PDTC) and an AP-1 inhibitor Curcumin in BALB/c mice. The alterations in these molecules were assessed by TF activity/expression and the expression of adhesion molecules (VCAM-1, ICAM-1, and E-selectin) and proinflammatory cytokines (IL-1β, IL-6, and TNF-α) in the carotid artery, aorta and peritoneal macrophages *in vivo*.

## Materials and Methods

### Mice and Chemicals

BALB/c mice weighing approximately 22 g (8–10 weeks of age) were purchased from Comparative Medicine Centre of Yangzhou University (Yangzhou, China). All animals were housed in the Laboratory Animal Research Center of Jiangsu University. All animal experiments were approved by the Laboratory Animal Administration Committee of Jiangsu University and consistent with the Guide for the Care and Use of Laboratory Animals published by the US National Institutes of Health.

Pyrrolidinedithiocarbamic acid (PDTC) (Sigma, Saint Louis, MO, USA) was dissolved in sterilized PBS and Curcumin (Sigma, USA) was dissolved in olive oil, aliquoted and stored at -20°C for the experiments *in vivo*.

### Preparation of IgGs

Polyclonal anti-β_2_GPI antibodies were purified from sera of New Zealand rabbits immunized with human β_2_GPI. Control antibodies (NR-IgG) from normal rabbits were purified by Protein G Sepharose columns. Total IgG containing the aPL antibodies (IgG-APS) from patients with primary APS was purified using Protein G Sepharose columns. In addition, the sera from APS patients displayed high titers of anti-β_2_GPI antibodies (≧66.9 SGU/mL). Ethical approval was granted by the Institutional Review Board of the Affiliated Hospital of Jiangsu University. All IgG samples and reagents were subjected to Detoxi-GelTM (Pierce, Rockford, IL, USA) to remove endotoxin contamination (<0.03 EU/ml) by the Limulus amebocyte lysate assay (ACC, Falmouth, MA, USA).

### Isolation of mouse carotid artery, aorta and peritoneal macrophages

The male BALB/c mice (9–10 animals/group) were injected by intraperitoneal injection (i.p.) with 500 μg of NR-IgG or anti-β_2_GPI or APS-IgG twice (at 0 and 48 h). In some experiments, mice were treated with PDTC (100 mg/kg, once a day) in phosphate-buffered saline (PBS) by i.p. or Curcumin (50 mg/kg, once a day) in olive oil by oral gavage daily for 3 consecutive days at 2 h before the IgG injections. BALB/c mice were anesthetized with pentobarbital (50 mg/kg, i.p.) and exposed by surgical procedures. Pieces of approximately 5 mm of carotid arteries were dissected from both sides in each mouse and were collected in a Tris (hydroxymethyl) aminomethane-buffered saline (TBS)/0.1% Triton X-100 buffer containing heparin and homogenized. Homogenates of pooled carotid artery from four mice in each group were washed once with TBS-0.1% Triton X-100 containing heparin and twice with TBS-0.1% Triton X-100, and used for experiments. Then aortas from six mice were collected in a TBS/0.1% Triton X-100 buffer containing heparin. Hereafter, vascular membranes were completely peeled under stereo microscope and the remaining blood vessels were used for experiments. Finally, isolation of peritoneal macrophages was done in the animals immediately after the surgical procedures and after they were sacrificed. Peritoneal macrophages of the mice were obtained by flushing the peritoneal cavity of the mice with 10 mL of PBS solution for 5 min. The macrophages were suspended in RPMI 1640 (Gibco BRL, Grand Island, NY, USA) without fetal bovine serum (FBS) (Hyclone, Logan, UT, USA) solution after centrifuged at 1000 rpm, 4°C, for 10 min. The 2.0 × 10^6^ cells/well was incubated in 6 well flat-bottomed plates at 37°C and 5% CO_2_ in a humidified cell incubator. After 30 min, nonadherent cells were removed and the remaining cells were washed twice with PBS, were then used for experiments.

### Western blotting analysis

The aortas and macrophages were collected and lysed in 200 μL RIPA buffer containing 20 mM Tris-HCl (pH7.5), 150 mM NaCl, 1% Triton X-100, 1 mM PMSF, 2.5 mM EDTA, and homogenized by sonication. The lysates were centrifuged at 10,000 rpm for 20 min (Kubota 6930, Tokyo, Japan). Protein samples underwent 12% SDS-PAGE and were then transferred onto a polyvinylidene difluoride (PVDF) membrane (Bio-Rad, Hercules, CA, USA), which was blocked with 5% fat-free milk in TBST buffer (20 mmol/L Tris-HCl, 137 mmol/L NaCl and 0.1% Tween 20) for 2 h at room temperature (RT), then incubated with primary antibodies against NF-κB p65, p-NF-κB p65, c-Jun, p-c-Jun, IL-6 and TNF-α (1:1000, Cell Signaling Technology, Beverly, MA, USA), ICAM-1, VCAM (both at 1:1000, Bioworld, St. Louis, MN, USA), E-selectin and TF (1:1000, Abcam, Cambridge, UK), IL-1β (1:200, Santa Cruz, CA, USA) and β-actin (1:1000, Proteintech Group, Chicago, IL, USA) in TBST buffer overnight at 4°C, then washed and incubated with secondary antibodies for 1 h at 37°C. Finally, the immunoblots were visualized using ECL Western blotting detection reagents (GE Healthcare, Buckinghamshire, UK).

### Real-time quantitative polymerase chain reaction (RT-qPCR) amplification

The aortas and macrophages were collected and homogenized by sonication in 200 μL TRIzol (Invitrogen, Carlsbad, CA, USA). Total RNA was isolated by using TRIzol according to the manufacturer’s protocol. Oligo dT-primers were used for reverse transcription with 1 μg of total RNA in a 10 μL reaction volume (TOYOBO Bio-Technology, Osaka, Japan; 2720 Thermal Cycler). The levels of target mRNA were performed by qPCR using SYBR Green I dye (Takara Biotec, Kyoto, Japan). The primers pairs used for PCR were shown in Table 1. Results were expressed as fold differences relative to the level of *GAPDH* using the 2^-ΔΔCT^ method.

**Table 1:** Primers used for real-time qPCR analysis.

Gene	Oligonucleotide primer sequences (5’–3’)	Amplicon size (bp)	Annealing (°C)
*TF*	F:TCAAGCACGGGAAAGAAAAC R:CTGCTTCCTGGGCTATTTTG	137	60
*E-selectin*	F:ATAACGAGACGCCATCATGC R:TGTCCACTGCCCTTGTGC	191	58.5
*ICAM-1*	F:CTCACTTGCAGCACTACGG R:TTCATTCTCAAAACTGACAGGC	138	59.7
*VCAM-1*	F:GCCACCCTCACCTTAATTGCT R:GCACACGTCAGAACAACCGAA	188	61
*IL-1β*	F:GCTGCTTCCAAACCTTTGACC R:AGCCACAATGAGTGATACTGCC	110	56
*IL-6*	F:GACTTCCATCCAGTTGCCTT R:ATGTGTAATTAAGCCTCCGACT	150	59.3
*TNF-α*	F:ATTATGGCTCAGGGTCCAAC R:GACAGAGGCAACCTGACCAC	197	60.4
*GAPDH*	F:GGCATTGCTCTCAATGACAA R:TGTGAGGGAGATGCTCAGTG	200	58

### Immunofluorescence

Mouse peritoneal macrophages were harvested and cultured in RPMI 1640 supplemented with 1% penicillin/streptomycin in 24 well flat-bottomed plates at 37°C and 5% CO_2_ in a humidified cell incubator. After 30 min, non-adherent cells were removed by washing. The adherent macrophages were fixed in 4% paraformaldehyde for 20 min at 37°C, and permeabilized with ice-cold 0.3% Triton X-100 for 10 min at room temperature (RT), and blocked in PBS containing 5% bovine serum albumin (BSA) for 1 h at RT. The cells were then incubated overnight at 4°C with TF (1:200; Abcam), IL-1β (1:50; Santa Cruz), IL-6 (1:200; Cell Signaling Technology), and TNF-α antibodies (1:200; Cell Signaling Technology), respectively. Subsequently, cells were incubated with Cy3-conjugated goat anti-rabbit IgG (1:200, Santa Cruz) for 1 h at room temperature. Nuclear counterstaining was performed with DAPI for 10 min at RT. Immunofluorescence images were acquired using A Zeiss fluorescence microscope with 20×. All image acquisition parameters were kept constant throughout all experiments.

### TF activity measurement

TF activity in mouse carotid artery homogenates and peritoneal macrophages was investigated in our study. The carotid artery and peritoneal macrophages resuspended in 50 μL of a TBS/0.1% Triton X-100 buffer containing heparin and sonicated. TF activity was determined as factor X activation by TF/VIIa complex by utilizing a commercial chromogenic assay (Assaypro, Greenwich, CT, USA). The concentration of generated factor Xa was calculated in pM/10^6^ cells in peritoneal macrophages and pM/mg protein in carotid artery homogenates, respectively.

### Statistical analysis

All experimental points were performed in triplicate or quadruplicate, and all assays were repeated a minimum of 3 times. Normally distributed variables were expressed as means ± standard deviation (SD). Differences between control and experimental conditions were assessed using the Student’s two-tailed t test for paired samples. For multiple group comparisons, we used ANOVA with Dunnett’s post test. All statistical analyses were performed using SPSS statistical software package version 20.0 (SPSS, Chicago, IL, USA). Statistical significance was defined as *p*<0.05.

## Result

### IgG-APS and anti-β_2_GPI induce phosphorylation of NF-κB and AP-1 in mouse peritoneal macrophages

**Fig 1:**
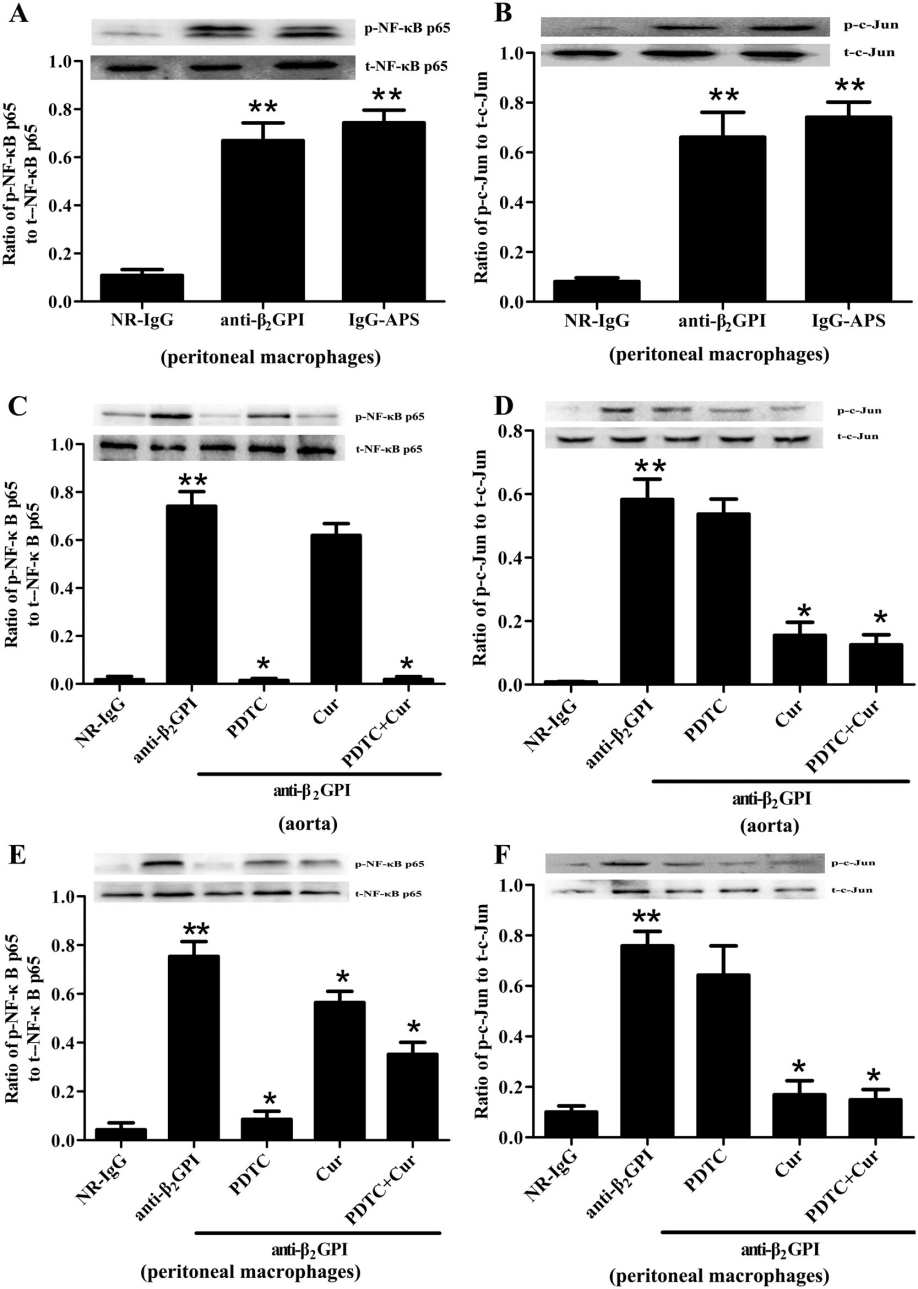
The effects of PDTC and Curcumin on anti-β_2_GPI-mediated NF-κB and AP-1 phosphorylation. BALB/c mice (4 per group) were injected with NR-IgG (500 μg) or IgG-APS (500 μg) or anti-β_2_GPI (500 μg) in the presence or absence of PDTC (100 mg/kg, once a day) or/and Curcumin (50 mg/kg, once a day), as described in Materials and Methods. Aorta homogenates (C-D) and peritoneal macrophages lysates (A-B, E-F) were collected for analyzing the phosphorylation levels of NF-κB p65 and c-Jun by western blotting using specific NF-κB p65, p-NF-κB p65, c-Jun, p-c-Jun, and control β-actin antibodies, respectively. Shown are the pooled data of three separate experiments with similar results. **Statistically significant difference from NR-IgG group (*p*<0.05) *statistically significant difference from the anti-β_2_GPI group (*p*<0.05).

Our previous studies demonstrated that NF-κB and c-Jun/AP-1 are involved in anti-β_2_GPI/β_2_GPI-induced tissue factor expression in monocytes [17], but these results have not been validated *in vivo*. Therefore, we firstly measured the phosphorylation of NF-κB and c-Jun/AP-1 in the peritoneal macrophages from the male BALB/c mice injected with NR-IgG, or anti-β_2_GPI, or IgG-APS. As shown in Fig 1A and 1B, the phosphorylation of NF-κB p65 and c-Jun/AP-1 was markely elevated in the peritoneal macrophages from BALB/c mice after anti-β_2_GPI or IgG-APS treatment, compared to control mice injected with NR-IgG. The phosphorylation levels of NF-κB p65 and c-Jun in mice injected with anti-β_2_GPI were almost similar to those in mice injected with anti-β_2_GPI or IgG-APS.

### PDTC and Curcumin abrogate the phosphorylation of NF-κB and AP-1 in mice treated with anti-β_2_GPI

The anti-β_2_GPI-induced NF-κB and c-Jun/AP-1 phosphorylations were further validated by using specific NF-κB and c-Jun/AP-1 inhibitors *in vivo*. BALB/c mice were pretreated with NF-κB inhibitor PDTC (100 mg/kg, once a day) by i.p. or AP-1 inhibitor Curcumin (50 mg/kg, once a day) by oral gavage for 3 consecutive days at 2 h before anti-β_2_GPI injections in subsequent experiments. Then we harvested aorta and peritoneal macrophages from experimental animals. Compared with the mice treated with anti-β_2_GPI alone, PDTC treatment dramatically inhibited the anti-β_2_GPI-induced phosphorylation of NF-κB p65 in the aorta (Fig 1C) and peritoneal macrophages (Fig 1E). Curcumin treatment also obviously decreased the anti-β_2_GPI-induced phosphorylation of NF-κB p65 in peritoneal macrophages (Fig 1E), and slightly decreased but not statistically significant in the aorta (*p*>0.05). Futhermore, the anti-β_2_GPI-induced phosphorylation of c-Jun in the aorta and peritoneal macrophages was inhibited dramatically by Curcumin (Fig 1D and 1E). However, the levels of total NF-κB p65 and c-Jun in the aorta and peritoneal macrophages from anti-β_2_GPI-treated mice were not affected by PDTC and/or Curcumin. In addition, combination of PDTC and Curcumin didn’t show amplified effects on inhibiting the phosphorylation of NF-κB p65 and c-Jun/AP-1.

### PDTC and Curcumin inhibit TF expression and activity in mice treated with anti-β_2_GPI

**Fig 2:**
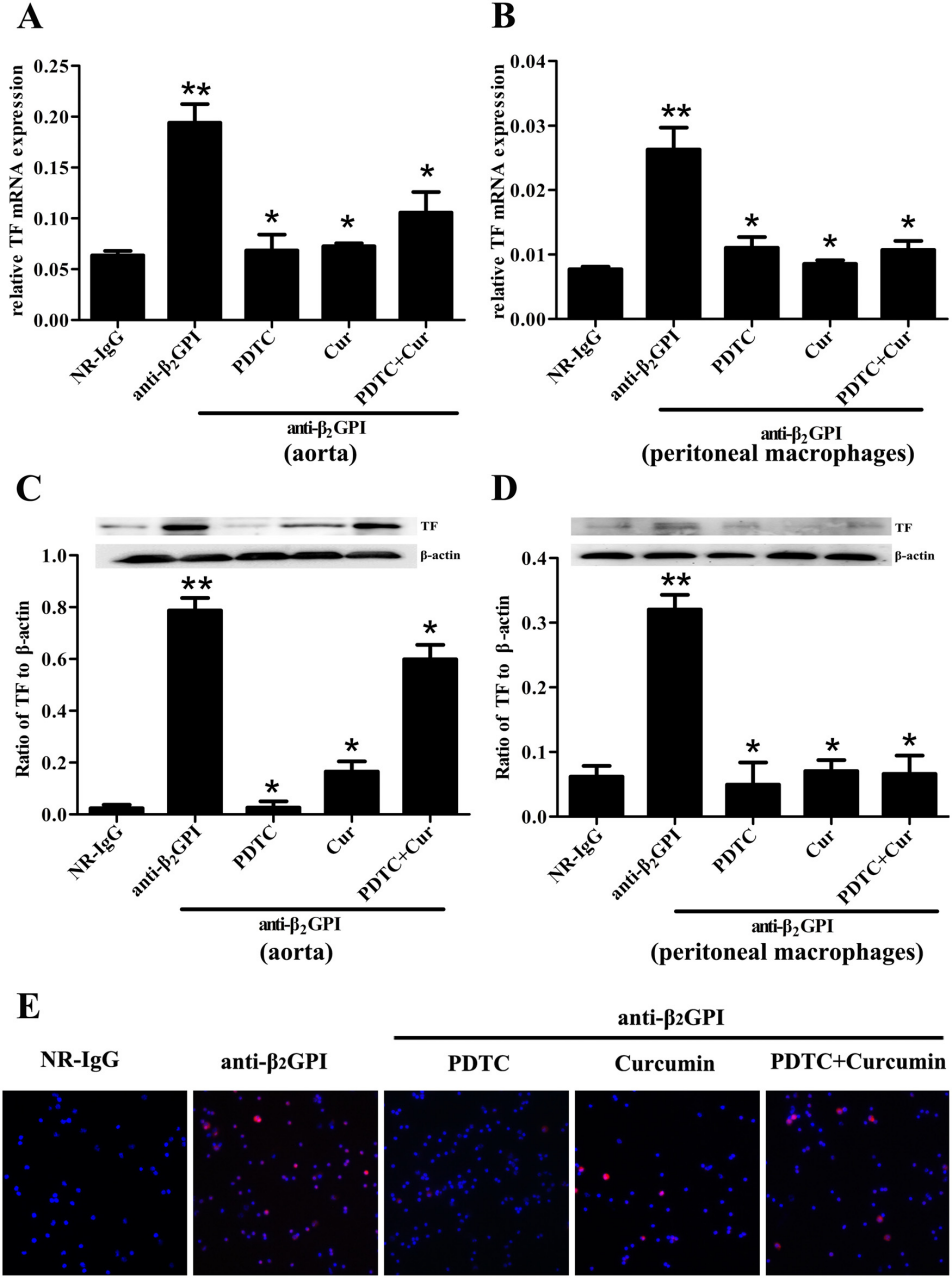
Anti-β_2_GPI-induced TF expression in mouse is diminished by PDTC or/and Curcumin. BALB/c mice (6 per group) were injected with NR-IgG (500 μg) or anti-β_2_GPI (500 μg) in the presence of PDTC (100 mg/kg, once a day) or/and Curcumin (50 mg/kg, once a day), as described in Materials and Methods. Total RNAs and protein were extracted from Aorta homogenates and peritoneal macrophages lysates. The expression of *TF* mRNA in aorta homogenates (A) and peritoneal macrophage lysates (B) were assessed using RT-qPCR. The expression of TF protein in aorta homogenates (C) and peritoneal macrophage lysates (D) were respectively measured by western blotting. TF expression in peritoneal macrophage was determined by immunolabeling and fluorescence microscopy (E). Representative images of TF expression on the macrophage surface of different treatment groups. The red fluorescence represents surface TF immunoreactivity, whereas the blue color confirms the presence of cells stained with DAPI reagent (original magnification ×200). **Statistically significant difference from NR-IgG group (*p*<0.05) *statistically significant difference from the anti-β_2_GPI group (*p*<0.05).

It has been widely reported that aPL can stimulate the upregulation of TF expression and activity in vascular endothelial cells and monocytes, thereby leading to increased thrombosis [10, 22–24]. To demonstrate the important roles of NF-κB and c-Jun/AP-1 in anti-β_2_GPI-induced TF expression and its activity *in vivo*, we sought to examine the effect of PDTC and Curcumin on TF expression in anti-β_2_GPI-treated mice. Aorta and peritoneal macrophages were obtained from BALB/c mice after different treatments, and were used to detect expression of TF mRNA and protein by RT-PCR, Western blotting and immunofluorescence. We found that anti-β_2_GPI could significantly upregulate the expression of *TF* mRNA (Fig 2A and 2B) and protein (Fig 2C and 2D) in aorta and peritoneal macrophages compared with NR-IgG treated mice (*p*<0.05). It seemed that anti-β_2_GPI-caused expressions of TF protein and mRNA in peritoneal macrophage are lower than that in the aorta (Fig 2A–2D). Importantly, the pretreatment with PDTC and/or Curcumin could significantly attenuate anti-β_2_GPI-induced TF expression in aorta and peritoneal macrophages (Fig 2A–2D) (*p*<0.05). Among all treatment groups, PDTC pretreatment showed the strongest inhibitory effect on the expression of anti-β_2_GPI-induced *TF* mRNA and protein in aorta and peritoneal macrophages. Furthermore, similar results of TF protein expression in peritoneal macrophages were observed by immunofluorescence (Fig 2E).

**Fig 3:**
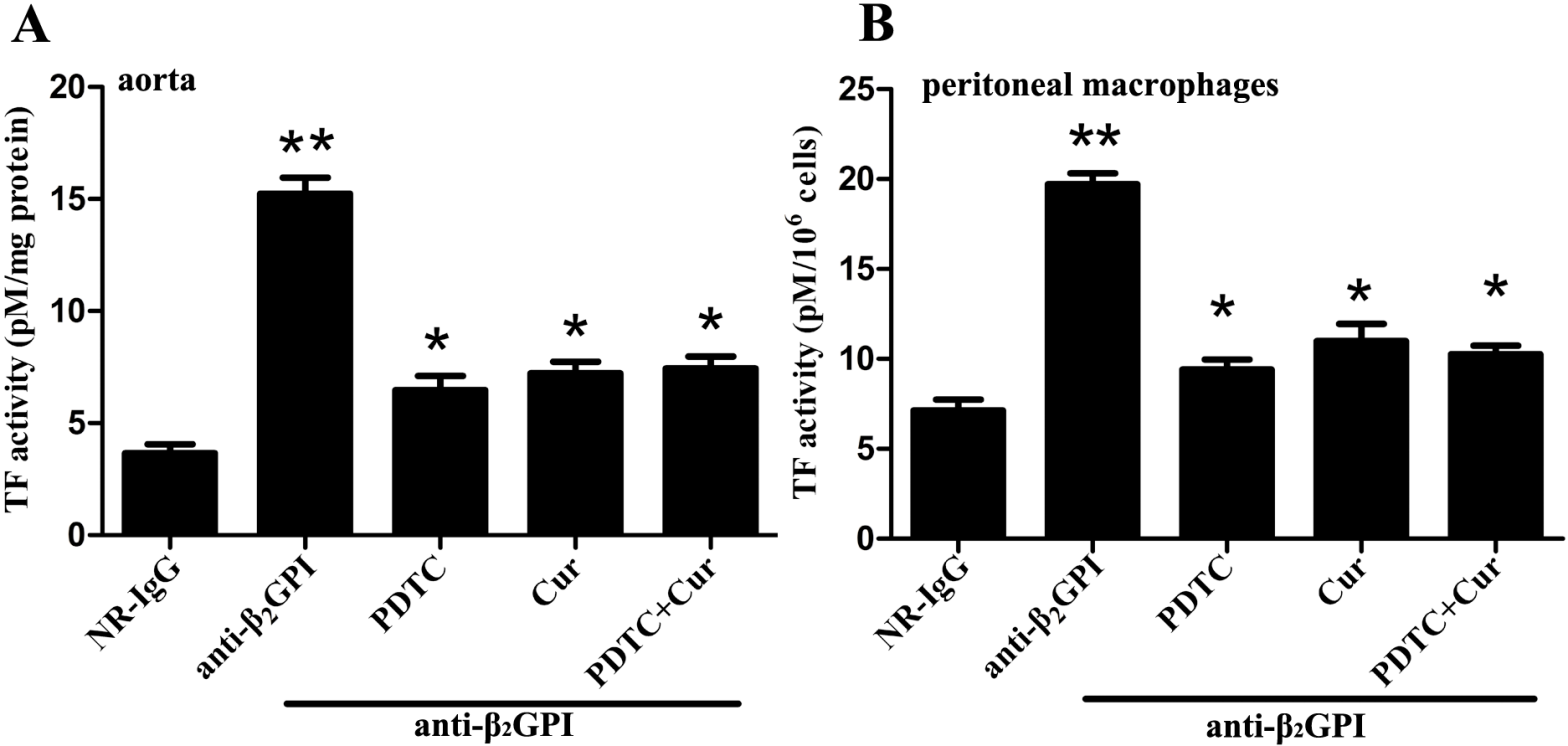
Anti-β_2_GPI-induced TF activity in mouse is abrogated with PDTC or/and Curcumin treatment. BALB/c mice (4 per group) were injected with NR-IgG (500 μg) or anti-β_2_GPI (500 μg) in the presence of PDTC (100 mg/kg, once a day) or/and Curcumin (50 mg/kg, once a day), as described in Materials and Methods. The carotid arteries (A) and peritoneal macrophages (B) were harvested from mice at 72 h after the frist injection, and TF activity in homogenates was determined by a commercial kit as described in Materials and Methods. Shown are representative of three independent experiments. **Statistically significant difference from NR-IgG group (*p*<0.05) *statistically significant difference from the anti-β_2_GPI group (*p*<0.05).

Next, we examined the influence of PDTC and Curcumin on anti-β_2_GPI-induced TF activity *in vivo*. TF activities in carotid artery homogenates (Fig 3A) and peritoneal macrophage lysates (Fig 3B) from anti-β_2_GPI-treated mice were significantly higher than those in NR-IgG-treated mice (*p*<0.05 vs NR-IgG). The TF activity in macrophage and aorta was almost similar (Fig 3). However, PDTC and Curcumin pretreatments significantly blocked TF activity in carotid artery homogenates (Fig 3A) and peritoneal macrophage lysates (Fig 3B) from anti-β_2_GPI-treated mice (*p*<0.05, *vs*. anti-β_2_GPI group). However, the combined treatments of PDTC and Curcumin had no synergistic effects on TF activity, consistent with the trend of NF-κB and c-Jun/AP-1 phosphorylation.

### PDTC and Curcumin attenuate anti-β_2_GPI-induced expression of E-selectin, ICAM-1 and VCAM-1

**Fig 4:**
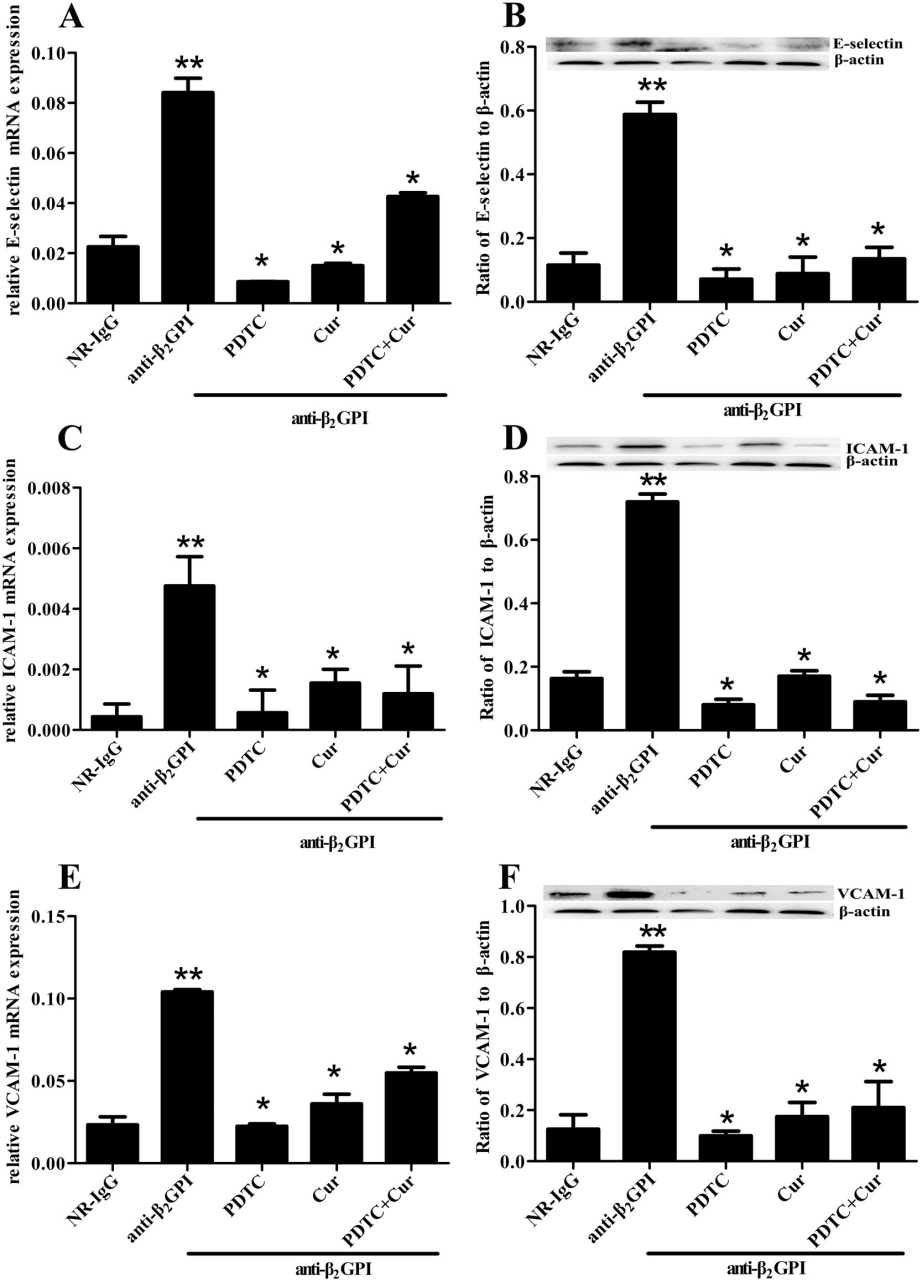
Inhibition of anti-β_2_GPI-induced E-selectin, ICAM-1 and VCAM-1 expression by PDTC or/and Curcumin. BALB/c mice (6 per group) were injected with NR-IgG (500 μg) or anti-β_2_GPI (500 μg) in the presence of PDTC (100 mg/kg, once a day) or/and Curcumin (50 mg/kg, once a day), as described in Materials and Methods. Total RNAs were obtained from frozen aortic wall. The relative mRNA levels of *E-selectin* (A), *ICAM-1* (C) and *VCAM-1* (E) in aorta were respectively detected by RT-qPCR. Aorta homogenates were collected for analyzing the protein expression of E-selectin (B), ICAM-1 (D) and VCAM-1 (F) by Western blotting using specific E-selectin, ICAM-1, VCAM-1 and control β-actin antibodies, respectively. Shown are the pooled data of three separate experiments with similar results. **Statistically significant difference from NR-IgG group (*p*<0.05) *statistically significant difference from the anti-β_2_GPI group (*p*<0.05).

We further examined whether PDTC and Curcumin can attenuate anti-β_2_GPI—induced endothelial cell (EC) activation by measuring the expression of E-selectin, ICAM-1 and VCAM-1 in aortic homogenates. Relative mRNA expression of *E-selectin*, *ICAM-1* and *VCAM-1* in aortas is shown in Fig 4A, 4C and 4E. Anti-β_2_GPI injection induced a significant increase in relative mRNA expression of *E-selectin*, *ICAM-1* and *VCAM-1* in aortic homogenates (*p*<0.05, *vs*. NR-IgG). The elevated expression of *E-selectin*, *ICAM-1* and *VCAM-1* mRNA in aortic homogenates from anti-β_2_GPI-treated mice was significantly blocked by PDTC and/or Curcumin pretreatments, in which PDTC showed the strongest inhibitory effects (*p*<0.05, *vs*. anti-β_2_GPI group). Interestingly, the combination of PDTC and Curcumin treatment didn’t show enhanced inhibitory effects. Consistent with mRNA expression, the similar changes in protein expression of E-selectin, ICAM-1 and VCAM-1 were observed in aortic homogenates from anti-β_2_GPI-treated mice after PDTC and/or Curcumin pretreatments (Fig 4B, 4D and 4F).

### PDTC and Curcumin attenuate anti-β_2_GPI-induced expression of IL-1β, IL-6 and TNF-α

**Fig 5:**
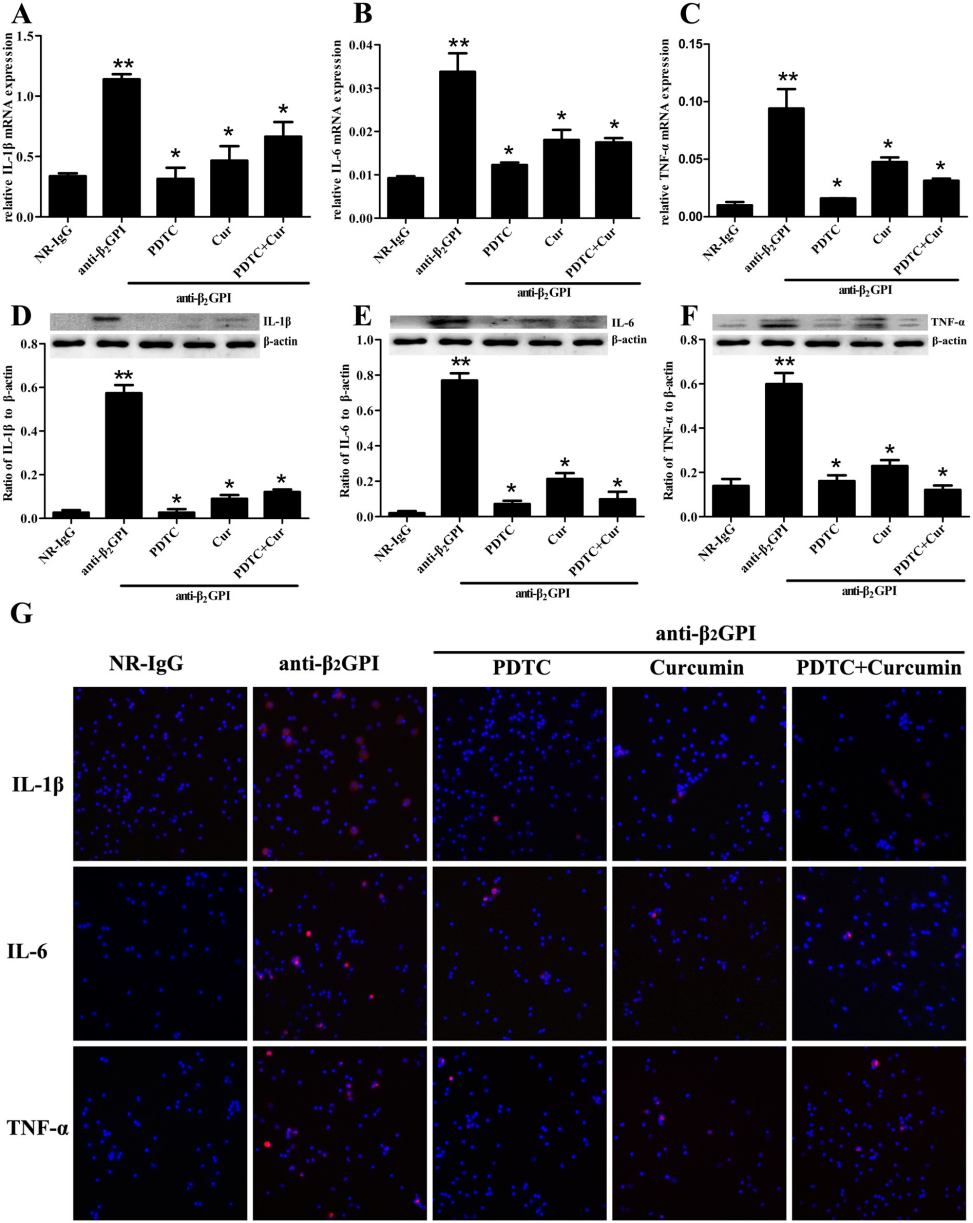
The effects of anti-β_2_GPI antibodies on IL-1β, IL-6 and TNF-α expression by PDTCor/ and Curcumin in mice. BALB/c mice (6 per group) were injected with NR-IgG (500 μg) or anti-β_2_GPI (500 μg) in the presence of PDTC (100 mg/kg, once a day) or/and Curcumin (50 mg/kg, once a day), as described in Materials and Methods. Total RNAs was prepared from mouse peritoneal macrophage, and the relative mRNA levels of *IL-1β* (A), *IL-6* (B) and *TNF-α* (C) were respectively measured by RT-qPCR. Peritoneal macrophage lysates from mice of different treatment were collected for detection of IL-1β (D), IL-6 (E) and TNF-α (F) by using specific IL-1β, IL-6, TNF-α and control β-actin antibodies, respectively. (G) Representative images were shown of IL-1β, IL-6 and TNF-α expression on the peritoneal macrophage among the different treatment groups. The red fluorescence indicates IL-1β, IL-6 and TNF-α immunoreactivity, whereas the blue color represents cell nuclei stained with DAPI reagent (original magnification ×200). Shown are the pooled data of three separate experiments with similar results. **Statistically significant difference from NR-IgG group (*p*<0.05) *statistically significant difference from the anti-β_2_GPI group (*p*<0.05).

We also determined whether PDTC and Curcumin abrogated anti-β_2_GPI—induced expression of IL-1β, IL-6 and TNF-α in peritoneal macrophages by RT-PCR, Western blotting and immunofluorescence staining. We found that anti-β_2_GPI could induce a significant upregulation of relative mRNA levels of *IL-1β*, *IL-6* and *TNF-α* in peritoneal macrophages (Fig 5A–5C, *p*<0.05, *vs*. NR-IgG). However, the upregulated mRNA levels of *IL-1β*, *IL-6* and *TNF-α* in the peritoneal macrophages from anti-β_2_GPI-treated mice were significantly attenuated by pretreating the mice with PDTC and/or Curcumin, in which PDTC showed the strongest inhibitory effects (Fig 5A–5C, *p*<0.05, *vs*. anti-β_2_GPI group). Curcumin could significantly restrain IL-1β expression, but not completely inhibit upregulation of IL-6 and TNF-α in the peritoneal macrophages from anti-β_2_GPI-treated mice. Moreover, the combination of PDTC and Curcumin pretreatments didn’t show enhanced inhibitory effects. Western blotting results showed that the elevated protein levels of IL-1β, IL-6 and TNF-α in peritoneal macrophage lysates from anti-β_2_GPI-treated mice were obviously abrogated in the presence of PDTC and/or Curcumin (Fig 5D–5F). Interestingly, the inhibitory effect of Curcumin on anti-β_2_GPI induced expression of these inflammatory molecules seemed a little weaker than that of PDTC, but the combined treatments of PDTC and Curcumin didn’t show enhanced inhibitory effects (Fig 5D–5F). Immunofluorescence staining results (Fig 5G) showed similar expression pattern to those of IL-1β, IL-6 and TNF-α proteins detected by Western Blot (Fig 5D–5F).

## Discussion

It is widely accepted that anti-β_2_GPI antibodies play a pathogenic role in APS and have been shown to induce activation of ECs, *in vitro* and *in vivo*, which may contribute to hypercoagulability in APS patients [12, 25–26]. Thrombin is correlated with the overall functional coagulation status of plasma in APS patients, and induced expression of connective tissue growth factors in rat vascular smooth muscle cells via the AP-1 pathway [27–28]. NF-κB may be involved in aPL/anti-β_2_GPI-induced expression of TF and adhesion molecules in ECs and monocytes *in vitro* [16, 29–30]. Our previous results demonstrated that both NF-κB and c-Jun/AP-1 are involved in anti-β_2_GPI/β_2_GPI-induced TF expression in monocytes [17]. In the present study, we show for the first time that NF-κB and c-Jun/AP-1 are also involved in anti-β_2_GPI-induced expression of prothrombotic and proinflammatory molecules *in vivo*, and that these effects can be attenuated by PDTC (a specific inhibitor of NF-κB) [31] and Curcumin (a potent inhibitor of AP-1) [32].

Although the important role of NF-κB in aPL/anti-β_2_GPI-induced pathogenic mechanisms has been widely recognized, this study specifically addressed the functions of anti-β_2_GPI-induced NF-κB and c-Jun/AP-1 activation *in vivo*. Interestingly, both IgG-APS from APS patients and polyclonal rabbit anti-human anti-β_2_GPI antibodies could activate a similar degree of NF-κB p65 and c-Jun/AP-1 phosphorylation in the peritoneal macrophages of BALB/c mice. These findings are consistent with our previous *in vitro* experimental data in monocytic cell line and THP-1 cells [17]. Hence, the polyclonal anti-β_2_GPI antibodies were used in subsequent experiments. Furthermore, a specific NF-κB inhibitor PDTC and an AP-1 inhibitor Curcumin were administrated to the mice for a relatively short period of time (72 h). PDTC and Curcumin could markedly attenuate anti-β_2_GPI-induced activation of NF-κB and c-Jun/AP-1 in the aorta and peritoneal macrophages respectively. Meanwhile, Curcumin alone also showed a significantly inhibitory effect on anti-β_2_GPI-induced NF-κB phosphorylation although its inhibitory effect was obviously weaker than that of PDTC. Possible reason is that Curcumin functions are cell specific and also functions on NF-κB. For example, Curcumin can inhibit LPS-induced inflammation by suppressing nuclear translocation of NF-κB in rat vascular smooth muscle cells [33]. It was also reported that Curcumin could inhibit the activation of NF-κB and AP-1 in aortic endothelial cells [34].

Previous studies demonstrated that anti-β_2_GPI-dependent induction of TF activity and expression in circulating blood monocytes and vascular endothelium are associated with the hypercoagulability in APS [24, 35]. In this study, we observed that anti-β_2_GPI-induced TF protein and mRNA expression in mouse peritoneal macrophage are lower than that in the aorta, but TF activity in macrophage and aorta was almost similar. The possible reason is because TF is mainly expressed by vascular cells, such as monocytes and endothelial cells. In addition, not all of expressed TF is active, and TF is normally expressed in cells or present in the blood in an encrypted form (inactive TF). This difference between TF protein and mRNA expression and TF activity limits inappropriate activation of the blood coagulation cascade [36]. Moreover, it is well established that NF-κB activation is required for aPL-induced TF upregulation *in vitro* [16, 37]. However, whether NF-κB and c-Jun/AP-1 are involved in prothrombotic effects of anti-β_2_GPI *in vivo* has been rarely investigated. Therefore, we hypothesize that PDTC or Curcumin could abrogate the procoagulant effects induced by anti-β_2_GPI antibodies *in vivo*. As expected, PDTC or Curcumin could significantly inhibit the upregulation of anti-β_2_GPI-induced TF activity in carotid artery homogenates and peritoneal macrophages, in which PDTC exerted the strongest inhibitory role. Consistent with these results, treatment with PDTC or Curcumin could also markedly inhibit anti-β_2_GPI-mediated expression of *TF* mRNA and protein in aortic homogenates and peritoneal macrophages. Likewise, PDTC showed the strongest inhibitory effect compared to other treatment groups. Interestingly, the combined treatments of PDTC and Curcumin didn’t show enhanced inhibitory effect on anti-β_2_GPI-induced TF expression and its activity *in vivo*. We speculate that both NF-κB and c-Jun/AP-1 play an indispensable role through their own target molecules in this process, since TF gene promoter contains two AP-1 sites and a NF-κB site [38]. More importantly, this is the first time to report that c-Jun/AP-1 can participate in anti-β_2_GPI-induced TF activity/expression *in vivo*. Therefore, we have sufficient reasons to believe that PDTC or Curcumin can decrease the thrombogenic effects of anti-β_2_GPI antibodies *in vivo* through inhibiting the activation of NF-κB and/or c-Jun/AP-1 signaling pathways.

Thrombosis is a devastating consequence and the most prominent clinical manifestation in APS, which may affect any organ in the body [39]. Nonetheless, the induction of an endothelial proinflammation and procoagulation by aPL has been widely accepted as a major pathogenic mechanism underlying the prothrombotic properties [40]. The aPL-induced ECs activation leads to loss of its anticoagulant properties and transformation to a pro-adhesive and procoagulant phenotype characterized by increased expression of adhesion molecules (E-selectin, ICAM-1, and VCAM-1) [5, 41–42]. Some recent studies reported that anti-β_2_GPI antibodies can induce signaling transduction through a multiprotein complex including annexin A2 and TLR4, in which TLR4 can induce the activation of TLR4/myeloid differentiation factor 88 (MyD88)-dependent pathway and NF-κB signaling in ECs and moncytes [41–44]. Indeed, our data demonstrated that anti-β_2_GPI antibodies upregulated the expression of ICAM-1, VCAM-1 and E-selectin in the aortic homogenates of mice. Moreover, these effects were significantly blocked in the presence of PDTC or Curcumin, in which PDTC showed the strongest inhibitory effect on the expression of ICAM-1, VCAM-1 and E-selectin in the aorta from anti-β_2_GPI-treated mice. We speculate that PDTC or Curcumin may inhibit anti-β_2_GPI-mediated expression of ICAM-1, VCAM-1 and E-selectin in the aorta by inhibiting the activation of NF-κB or/and c-Jun/AP-1, thus blocking the interaction of leukocytes and platelets with endothelium *in vivo*, and finally suppressing the inflammatory response and the procoagulant state.

It has reported that high levels of IL-1β, IL-6 and TNF-α are present in serum of APS patients, indicating the presence of a proinflammatory phenotype in the body [11, 45]. Our previous studies found that anti-β_2_GPI/β_2_GPI complexes promoted the expression of proinflammatory cytokines via TLR4/NF-κB signaling pathway *in vitro* [43, 46]. Here we further validate that NF-κB and c-Jun/AP-1 can modulate anti-β_2_GPI-induced expression of IL-1β, IL-6 and TNF-α in peritoneal macrophages from mice. The high expression of these inflammatory cytokines can be significantly abolished by pretreatment of PDTC or Curcumin before the anti-β_2_GPI injection. Moreover, PDTC can completely block their high expression, comparable to their in the NR-IgG-treated mice. These data indicate that NF-κB and c-Jun/AP-1 not only contribute to anti-β2GPI-induced procoagulant activity and expression of adhesion molecules, but also mediate proinflammatory responses *in vivo*. In the pathogenic mechanisms of APS, NF-κB may play a vital role while AP-1 may play a supporting role, thus there may be an ordinal relation between NF-κB and AP-1. Fujioka *et al*. reported that NF-κB regulates the expression of c-Fos and AP-1 activity through controlling the expression level of Elk-1 [47]. Interestingly, the combined treatments of PDTC and Curcumin sometimes showed even less inhibitory effects on anti-β2GPI-induced expression of prothrombotic and proinflammatory molecules in mouse compared with their individual treatment alone. Possible explanation is that simultaneous administration of PDTC and Curcumin may arise some kinds of side effects in mouse body, or some interactions between two inhibitors may impair their original role. So it’s not surprising that the combined pretreatments of PDTC and Curcumin did not show the enhanced inhibitory effects compared with their individual treatment alone, suggesting that separate administration of PDTC or Curcumin may achieve optimal inhibitory effects on their target molecules.

Recent accumulated evidence has demonstrated that a “two hit hypothesis” has been widely accepted to explain the clinical observation that thrombotic events occur occasionally in spite of the persistent presence of aPL. The hypothesis is that aPL (first hit) only increases the thrombophilic risk while the thrombosis will occur in the presence of another thrombophilic conditions (second hit), such as inflammation and infection [22, 48]. Our results provide solid evidence to support this “two hit hypothesis” in APS. Moreover, the activation of NF-κB and c-Jun/AP-1 is a crucial step in triggering the expression of prothrombotic and proinflammatory molecules during the pathogenesis of aPL-induced clinical manifestation. Additionally, the direct treatments for preventing thromboembolic events using antithrombotic medications and for modulating the immune response using immunotherapy have considerable side effects. However, there are also a lot of APS patients in whom aPL Abs are persistently present in the serum for a long period of time but thrombotic events only occur occasionally. Hence, anticoagulation is not always effective for all patients, otherwise it can increase a risk of hemorrhage. Furthermore, agents like PDTC or Curcumin may act directly on the putative “first hit” to ameliorate those effects, reduce the risk for developing clinical events when a “second hit” occurs. However, whether PDTC can be used for clinical patient still needs further investigation. It has been found that PDTC has some neurotoxicity and hepatotoxicity by generating some toxic compounds, including CS_2_ and pyrrolidine [49].

## Conclusion

Our results demonstrated that both NF-κB and c-Jun/AP-1 are involved in regulating anti-β_2_GPI-induced expression of prothrombotic and proinflammatory molecules in mouse model. NF-κB plays an indispensable role in aPL-mediated pathogenic effects in APS, while c-Jun/AP-1 is also involved in this process. PDTC and Curcumin treatment may be considered as a new approach to treat and/or prevent thrombosis in APS patients.
